# Multiple Loci within the Major Histocompatibility Complex Confer Risk of Psoriasis

**DOI:** 10.1371/journal.pgen.1000606

**Published:** 2009-08-14

**Authors:** Bing-Jian Feng, Liang-Dan Sun, Razieh Soltani-Arabshahi, Anne M. Bowcock, Rajan P. Nair, Philip Stuart, James T. Elder, Steven J. Schrodi, Ann B. Begovich, Gonçalo R. Abecasis, Xue-Jun Zhang, Kristina P. Callis-Duffin, Gerald G. Krueger, David E. Goldgar

**Affiliations:** 1 Department of Dermatology, University of Utah School of Medicine, Salt Lake City, United States of America; 2 Institute of Dermatology, Anhui Medical University, Hefei, China; 3 Department of Dermatology, The First Affiliated Hospital of Anhui Medical University, Hefei, China; 4 Key Laboratory of Dermatology (Anhui Medical University), Ministry of Education, Hefei, China; 5 Department of Genetics, Washington University School of Medicine, St. Louis, Missouri, United States of America; 6 Department of Dermatology, University of Michigan, Ann Arbor, Michigan, United States of America; 7 Celera, Alameda, California, United States of America; 8 Department of Biostatistics, University of Michigan, Ann Arbor, Michigan, United States of America; National Institute of Genetics, Japan

## Abstract

Psoriasis is a common inflammatory skin disease characterized by thickened scaly red plaques. Previously we have performed a genome-wide association study (GWAS) on psoriasis with 1,359 cases and 1,400 controls, which were genotyped for 447,249 SNPs. The most significant finding was for SNP rs12191877, which is in tight linkage disequilibrium with HLA-Cw*0602, the consensus risk allele for psoriasis. However, it is not known whether there are other psoriasis loci within the MHC in addition to *HLA-C*. In the present study, we searched for additional susceptibility loci within the human leukocyte antigen (HLA) region through in-depth analyses of the GWAS data; then, we followed up our findings in an independent Han Chinese 1,139 psoriasis cases and 1,132 controls. Using the phased CEPH dataset as a reference, we imputed the HLA-Cw*0602 in all samples with high accuracy. The association of the imputed HLA-Cw*0602 dosage with disease was much stronger than that of the most significantly associated SNP, rs12191877. Adjusting for HLA-Cw*0602, there were two remaining association signals: one demonstrated by rs2073048 (p = 2×10^−6^, OR = 0.66), located within *c6orf10*, a potential downstream effecter of TNF-alpha, and one indicated by rs13437088 (p = 9×10^−6^, OR = 1.3), located 30 kb centromeric of *HLA-B* and 16 kb telomeric of *MICA*. When HLA-Cw*0602, rs2073048, and rs13437088 were all included in a logistic regression model, each of them was significantly associated with disease (p = 3×10^−47^, 6×10^−8^, and 3×10^−7^, respectively). Both putative loci were also significantly associated in the Han Chinese samples after controlling for the imputed HLA-Cw*0602. A detailed analysis of *HLA-B* in both populations demonstrated that HLA-B*57 was associated with an increased risk of psoriasis and HLA-B*40 a decreased risk, independently of HLA-Cw*0602 and the *C6orf10* locus, suggesting the potential pathogenic involvement of *HLA-B*. These results demonstrate that there are at least two additional loci within the MHC conferring risk of psoriasis.

## Author Summary

Psoriasis (Ps) is a chronic inflammatory disease of the skin, affecting approximately 2% of Europeans. The *HLA-C* gene, located within the major histocompatibility complex (MHC) region on chromosome 6, is the major genetic determinant of psoriasis. However, multiple susceptibility genes within MHC are also hypothesized. Recently, we carried out a genome-wide association scan (GWAS) on psoriasis with 1,359 patients and 1,400 healthy controls, which identified seven psoriasis loci in the human genome and confirmed the effect of *HLA-C*. This dataset contains densely distributed genetic variations, single nucleotide polymorphisms (SNPs), which were then further analyzed in search for additional susceptibility genes within the MHC region. Using the SNP data, we imputed in all samples the *HLA-C* risk allele with high accuracy. Adjusting for the *HLA-C*, two additional loci, one near *C6orf10* and one near *HLA-B/MICA*, have significant associations with psoriasis, which were also observed in an independent Han Chinese dataset, suggesting that within the MHC there are at least three genes moderating susceptibility to psoriasis.

## Introduction

Psoriasis (Ps) is a relatively common, T cell-mediated, inflammatory skin disease. Ps is typically manifested as thickened scaly red plaques, characterized by epidermal hyperplasia, increased vascularity in the dermis, and infiltration of inflammatory cells into the dermis and epidermis [1],[2],[3]. Both genetics and environment play a role in the etiology of Ps. Although its pathogenic mechanism is not completely understood, investigations have strongly suggested that a susceptibility locus (PSORS1) located within the human leukocyte antigen (HLA) class I region on the short arm of chromosome 6, is the major genetic determinant of psoriasis [4]. However, the exact location of the PSORS1 gene had been controversial, due to the extended and complicated linkage disequilibrium (LD) pattern of the region [5]. Early studies had indicated the existence of two major PSORS1 locations suggested by various fine mapping studies, one a region ∼150 kb telomeric to *HLA-C* [6],[7],[8],[9], harboring *HCG27*, *POU5F1*, *TCF19*, *CCHCR1* and *CDSN*, and the other *HLA-C* itself or very close [10],[11]. Recently, a combined sequencing and haplotype mapping study found that within the 298 kb homologous region between the two proposed risk haplotypes, only *HLA-C* encoded variants unique to these haplotypes at the level of translated protein, which at the same time conferred increased risk of psoriasis, strongly suggesting that *HLA-C* is the Ps susceptibility gene, and excluded the telomeric region [12]. Similarly, two other family-based association studies, one in a white population and the other in a Chinese population, confirmed the direct involvement of *HLA-C* in psoriatic susceptibility [13],[14]. In terms of specific risk alleles, HLA-Cw*0602 has been consistently reported in numerous populations, while the results have been controversial for HLA-Cw*1203, showing modest positive association with psoriasis in some studies [14],[15], or no association [12],[16], or even significantly lower frequency in psoriasis patents than in controls in others [17].

Located 85 kb centromeric to *HLA-C*, *HLA-B* has also repeatedly exhibited association with psoriasis [18]. However, the over-represented B serotypes, B*13 and B*57, are in tight LD with HLA-Cw*0602 [19]; therefore, the association of *HLA-B* is thought to be attributable to *HLA-C*, or the HLA-Cw*0602 harboring haplotypes 13.1 (Cw6-B13) and 57.1 (Cw6-B57), which are named according to the B allele [20]. Likewise, genes located farther from *HLA-C* at the centromeric end, including *TNF-α* (tumor necrosis factor-alpha) [21],[22], *AGER* (receptor of advanced glycosylation end product-specific receptor) [23], *HLA-DRB1*, *HLA-DQA1* and *HLA-DQB1* [24],[25],[26],[27], have also been found to be associated with psoriasis. Although some of these genes are as far away from *HLA-C* as 1.38 megabases, the extended haplotype pattern of the HLA region still makes it probable that the association of these genes can be explained by LD with PSORS1, i.e. *HLA-C* [11],[28].

Other findings have suggested the existence of another susceptibility gene in the major histocompatibility complex (MHC) in addition to *HLA-C*. A study found that the octamer transcription factor-3 (*OTF3*, also named *POU5F1*) B allele was more prevalent in patients than in controls, even within the HLA-Cw*0602-negative subset of samples [29]. Moreover, less than 25 kb from this gene, two single nucleotide polymorphisms (SNPs) in the *SEEK1* (*PSORS1C1*) gene retained association with psoriasis upon stratification for HLA-Cw*0602 status (positive/negative) [30]. However, these analyses did not consider HLA-Cw*1203, nor did they account for the increased risk associated with HLA-Cw*0602 homozygotes [31]. Thus, analyses conditional on HLA-Cw*0602 only, or upon stratification on HLA-Cw*0602 positive/negative status, may not completely remove the confounding by *HLA-C*. Moreover, in contrast to the telomeric end, the centromeric end of *HLA-C* has rarely been investigated conditional on PSORS1.

Recently, within the framework of the Genetic Association Information Network (GAIN) we performed a multi-center collaborative genome-wide association study (GWAS), which identified seven Ps susceptibility loci at a genome-wide level of significance [32]. In this study, the most significantly associated SNP was rs12191877 (p = 3×10^−53^), which is in strong LD with HLA-Cw*0602 (r^2^ = 0.63). In addition to this SNP, other MHC SNPs that reached genome-wide significance spanned a 4 Mb region, centering on *HLA-C*. Considering the widely scattered physical locations of these associated SNPs, the density of immune or inflammation related genes, and the above-mentioned multiple-susceptibility-genes in this region hypothesized to be associated with psoriasis, we searched for other psoriasis loci in the GAIN dataset by examining the MHC region in more detail. First, we used the CEPH phased data as a reference to derive a method of determining the *HLA-C* genotypes based on the SNPs genotyped as part of the GAIN projects [33]. This was followed by a stepwise search for other susceptibility genes within the MHC region conditional on the predicted HLA-Cw*0602. The findings from these analyses were then replicated in an independent case-control dataset from a Chinese population. These results demonstrate that within the MHC region, there are at least two susceptibility loci for Ps in addition to *HLA-C*.

## Results

### Imputation of HLA-Cw*0602 and HLA-Cw*1203 is accurate

Using the phased HLA and SNP genotypes contained in the HapMap CEU panel and additional CEPH samples [33] as a reference set, we imputed in each GAIN subject the HLA-Cw*0602 and HLA-Cw*1203 genotypes, represented as the predicted number of HLA-Cw*0602 or HLA-Cw*1203 alleles. Since the SNP combinations used in the imputation were in complete linkage disequilibrium with the *HLA-C* allele of interest in the reference samples, the calculated uncertainties in the imputation only arose from haplotype reconstruction. In all GAIN samples, this uncertainty level was very low, indicated by the small average difference between the imputed and the most likely genotypes (0.002 and 0.0001 for HLA-Cw*0602 and HLA-Cw*1203, respectively). In comparison with true *HLA-C* genotypes obtained from direct sequencing in a subset of our samples (n = 420), there were no discrepancies observed for HLA-Cw*0602, and only 2 for HLA-Cw*1203, leading to a discordant rate of less than 0.5% (Table 1).

**Table 1:** Comparison of the imputed *HLA-C* genotypes with true genotypes.

HLA allele	Imputed number of alleles	−/−	−/+	+/+
Cw*0602	0	247	0	0
	0.805	0	1	0
	0.955	0	1	0
	0.991	0	2	0
	0.992	0	1	0
	0.993	0	3	0
	0.994	0	1	0
	0.995	0	1	0
	0.996	0	7	0
	0.997	0	6	0
	0.998	0	4	0
	0.999	0	13	0
	1	0	115	0
	1.992	0	0	1
	1.998	0	0	3
	2	0	0	3
Cw*1203	0	368	1	0
	0.991	0	2	0
	0.998	0	1	0
	0.999	0	7	0
	1	1	28	0
	2	0	0	2

### *HLA-C* is the major susceptibility gene within the MHC

**Figure 1:**
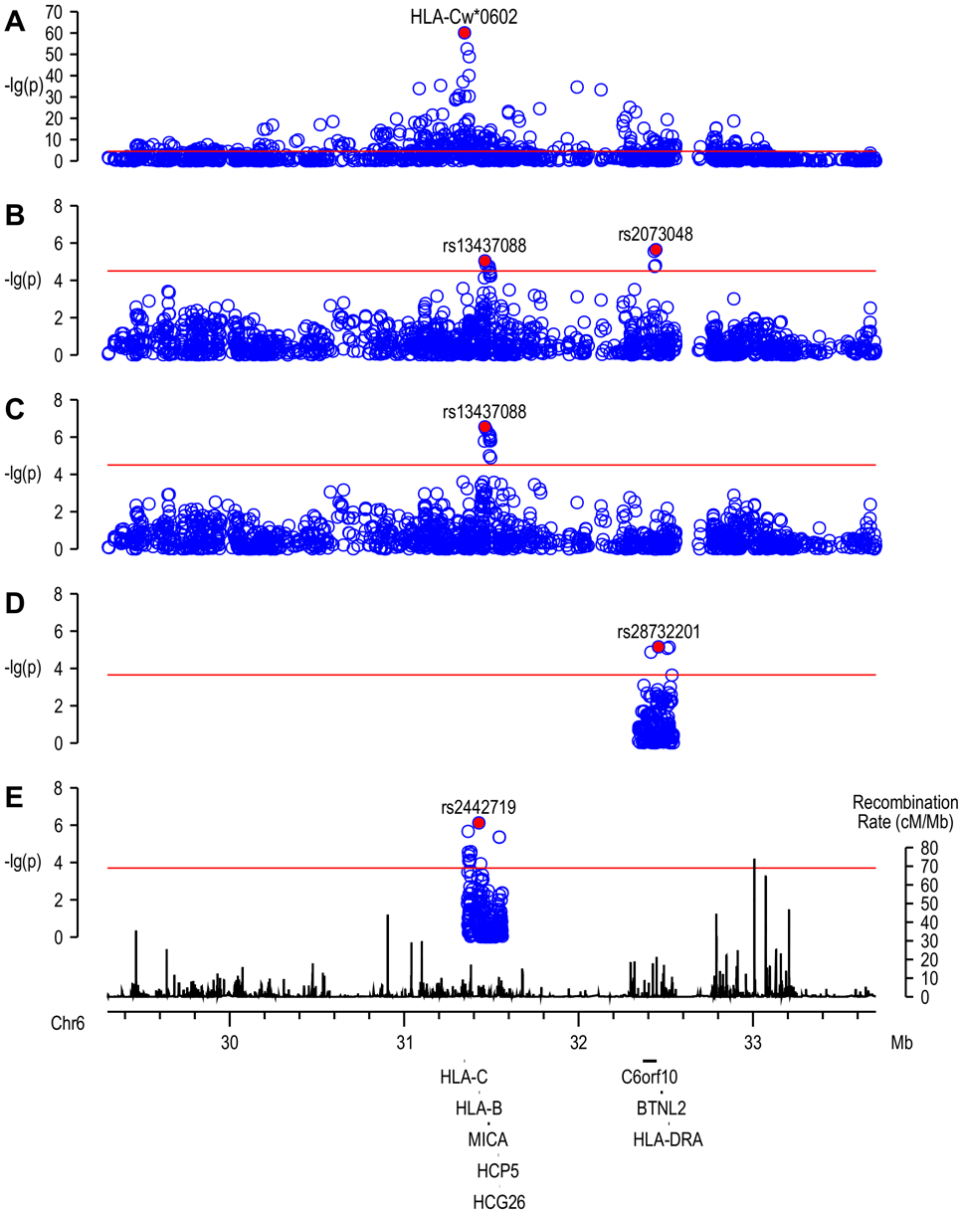
Association of MHC SNPs with psoriasis. (A) Trend test of MHC SNPs in the GAIN dataset; (B) Logistic regression adjusted for HLA-Cw*0602 in the GAIN dataset; (C) Logistic regression adjusted for HLA-Cw*0602 and rs2073048 in the GAIN dataset. (D) Logistic regression adjusted for HLA-Cw*0602 in the Chinese dataset; (E) Logistic regression adjusted for HLA-Cw*0602 and rs28732201 in the Chinese dataset. The red lines indicate the significance level by Bonferroni correction for the number of SNPs tested. The numbers of SNPs are 1593, 1593, 1591, 226, and 252 for (A–E), respectively.

In a logistic regression analysis, the imputed HLA-Cw*0602 allele was clearly associated with psoriasis. Both the significance level and the magnitude of association were higher than those observed for the most significant genotyped SNP, rs12191877 (p = 8×10^−61^ vs. p = 3×10^−53^; per allele OR = 3.85 [3.25–4.55] vs. OR = 2.92 [2.54–3.37]) (Figure 1A). Another suggested risk allele of *HLA-C*, HLA-Cw*1203 was significantly associated in logistic regression adjusted for HLA-Cw*0602 (p = 0.002, OR = 1.44 [1.14–1.81]), and in an analysis of the HLA-Cw*0602-negative subset of samples (p = 0.004, OR = 1.44 [1.12–1.85]). Imputation of other *HLA-C* major alleles did not show any association with psoriasis (data not shown). These provide further confirmation that *HLA-C* is the major susceptibility gene at the PSORS1 locus, and that HLA-Cw*0602 is the allele associated with the highest risk.

### *C6orf10* is associated with psoriasis

To examine the association of other loci within the MHC region with psoriasis, we applied logistic regression analyses to all other genotyped SNPs in the MHC region (from 29.3 Mb to 33.7 Mb on chromosome 6) adjusting for the imputed HLA-Cw*0602 genotype. As expected, given the patterns of linkage disequilibrium across this region, the levels of significance for association of the vast majority of SNPs dropped dramatically (Figure 1B). The top SNP in the unadjusted analyses, rs12191877, was no longer even nominally significantly associated with Ps after adjustment for HLA-Cw*0602.

However, as seen in Figure 1B, we observed other SNPs that remained significant after Bonferroni correction for multiple testing. The SNP exhibiting the highest significance level, rs2073048, is located at position 32.4 Mb within an open reading frame, *C6orf10*, 27 kb telomeric to *BTNL2* and 144 kb centromeric to *NOTCH4*. The minor allele (G) at this SNP had a frequency of 15% in controls and was associated with an adjusted odds ratio of 0.66 (95% confidence interval = [0.56–0.78], p = 2×10^−6^). To better understand the relationship between this locus and *HLA-C*, we examined the effect of rs2073048 in a stratified analysis in which strata were defined by carriage of HLA-Cw*0602. The test of homogeneity of effect between strata showed no evidence of heterogeneity (p = 0.78). In the subset of samples that does not contain an HLA-Cw*0602 allele, rs2073048 was still significantly associated with psoriasis (OR = 0.64 [0.53–0.78], p = 1×10^−5^). These results suggest that the association of this locus with psoriasis is independent of HLA-Cw*0602.

### *HLA-B/MICA* is associated with psoriasis

Another cluster of SNPs exhibiting significant p-values after Bonferroni correction in Figure 1B were between *HLA-B* and *MICA*. The most significant genotyped SNP of these, rs13437088, is located 30 kb centromeric of *HLA-B* and 16 kb telomeric of *MICA*. The minor allele of this SNP, T, had an allele frequency of 0.26 in controls, and was associated with an increased risk of psoriasis (OR = 1.32 [1.17–1.49], p = 9×10^−6^). In analyses adjusted for HLA-Cw*0602 and rs2073048 (Figure 1C), the association of this SNP was even stronger (OR = 1.38 [1.22–1.57], p = 3×10^−7^), suggesting that the observed association is not due to the LD with *HLA-C* or *C6orf10*.

In stratified analyses, there was no evidence of heterogeneity when samples were stratified by HLA-Cw*0602, rs2073048, or both (p = 0.21, 0.26, and 0.58, respectively), showing that the association of this locus is independent of *HLA-C* and of the *C6orf10* locus. When both of the newly identified putative susceptibility loci and HLA-Cw*0602 were included in a logistic regression model, each of them was significantly associated with disease (p = 3×10^−47^, 6×10^−8^ and 3×10^−7^, for HLA-Cw*0602, rs2073048 and rs13437088, respectively). Individuals carrying risk genotypes at *HLA-C*, rs2073048 and rs13437088 were estimated to be at a nine-fold increased risk of psoriasis compared to those carrying low risk genotypes at all three loci (Table 2).

**Table 2:** Psoriasis risk conferred by the three MHC loci in the GAIN dataset.

Cw*0602	rs13437088^a^	rs2073048^b^	Case (%)	Control (%)	O.R. [95%CI]	p-value
-	-	-	54 (4)	146 (10)	1	Ref.
-	-	+	306 (23)	518 (37)	1.60 [1.13–2.25]	0.007
-	+	-	86 (6)	189 (14)	1.23 [0.82–1.84]	0.31
-	+	+	277 (20)	308 (22)	2.43 [1.71–3.46]	5×10^−7^
+	-	-	25 (2)	19 (1)	3.56 [1.81–6.97]	0.0001
+	-	+	192 (14)	86 (6)	6.04 [4.04–9.03]	1×10^−19^
+	+	-	75 (6)	28 (2)	7.24 [4.24–12.4]	2×10^−14^
+	+	+	342 (25)	104 (7)	8.89 [6.07–13.0]	5×10^−33^

a ‘+’ denotes genotype CT/TT (presence of the risk allele T);

b ‘+’ denotes genotype AA (absence of the protective allele G).

To assess whether *HLA-B* is responsible for the association of rs13437088 with psoriasis risk, we imputed all *HLA-B* serotypes with CEPH population frequencies >5%, as well as those serotypes previously suggested to be associated with psoriasis (B*13, B*57 and B*58). In a logistic regression analysis adjusted for HLA-Cw*0602 and rs2073048, two B serotypes (B*40 and B*57) were significantly associated with psoriasis after Bonferroni correction for testing multiple serotypes: One additional copy of B*57 conferred a 1.7 fold elevated risk of disease, while B*40 was associated with a 40% reduced risk. In individuals who did not carry HLA-Cw*0602, B*40 remained significantly associated, while B*57 did not (Table 3). This can be explained by the fact that B*57 is in tight LD with HLA-Cw*0602, while B*40 is not in LD with HLA-Cw*0602 [19]. The SNP rs13437088 was in high LD with B*57 (D' = 1).

**Table 3:** Association of major *HLA-B* serotypes with psoriasis in the GAIN dataset.

HLA-B serotype	Adjusted O.R. [95%CI]^a^	Adjusted p-value^a^	Cw6 -/- O.R. [95%CI]^b^	Cw6 -/- p-value^b^
7	0.90 [0.75–1.07]	0.24	0.91 [0.74–1.10]	0.33
8	1.13 [0.93–1.37]	0.23	1.13 [0.91–1.38]	0.27
13	1.16 [0.83–1.64]	0.38	1.26 [0.47–3.37]	0.64
15	1.01 [0.81–1.26]	0.91	1.09 [0.86–1.39]	0.48
18	0.96 [0.71–1.30]	0.80	0.93 [0.68–1.29]	0.67
35	0.91 [0.74–1.13]	0.40	0.90 [0.71–1.14]	0.38
40	0.61 [0.47–0.79]	0.0002^c^	0.65 [0.49–0.87]	0.003^c^
44	0.85 [0.71–1.03]	0.09	0.77 [0.62–0.95]	0.01
57	1.66 [1.25–2.19]	0.0004^c^	2.92 [1.36–6.25]	0.006
58	1.22 [0.54–2.73]	0.64	1.09 [0.44–2.71]	0.85

a Adjusted for HLA-Cw*0602 and rs2073048;

b In the HLA-Cw*0602-negative subset of samples, adjusted for rs2073048;

c Significant after Bonferroni correction (p<0.005).

### Replication of both loci in a Han Chinese population

To further confirm our findings of the two novel loci for psoriasis within MHC, we tested them in an independent Han Chinese sample that was included in another GWAS of psoriasis [34]. Using the Han Chinese and Japanese HapMap data as a reference, HLA-Cw*0602 genotypes were imputed using strategies identical to those for the US sample. The predicted HLA-Cw*0602 allele was strongly associated with psoriasis (p = 1×10^−206^), which is quite comparable to the top SNP rs1265181 (p = 2×10^−208^) [34], whose genotypes were 99.6% identical to the predicted HLA-Cw*0602, showing that HLA-Cw*0602 is the main susceptibility allele within the PSORS1 region. On the other hand, HLA-Cw*1203 was not associated with psoriasis in this population, after adjustment for HLA-Cw*0602 or within the HLA-Cw*0602-negative subset of samples.

To assess the two loci we observed in the US samples, we first tested all genotyped SNPs within 100 kb of rs2073048, the first locus we identified in the US study. The results indicated several SNPs that were significantly associated, the most significant of which was the SNP rs28732201, which had a minor allele frequency of 0.01, and odds ratio of 2.85 [1.81–4.50] with a p-value of 7×10^−6^ (Figure 1D). This SNP is located between *C6orf10* and *BTNL2*, 11 kb upstream to the transcription start site of *C6orf10*, and 12 kb downstream to the transcription end site of *BTNL2*. After adjustment for both HLA-Cw*0602 and rs28732201, the locus of *HLA-B/MICA* (within 100 kb of the SNP identified in the GAIN dataset) also exhibited significant association, shown by the SNP rs2442719 (OR = 1.66 [1.36–2.03], p = 8×10^−7^, Figure 1E), located only 1 kb from the telomeric end of *HLA-B*. When HLA-Cw*0602, rs28732201 and rs2442719 were all included in a logistic regression model, they all remained significantly associated (p = 2×10^−102^, 1×10^−5^, and 8×10^−7^, respectively).

We also imputed the B*40 and B*57 serotypes in these Chinese samples and tested their associations with psoriasis controlling for HLA-Cw*0602. Interestingly, similar to the US data, B*57 was significantly associated with an increased risk of psoriasis (OR = 2.71 [1.81–4.06], p = 1×10^−6^), and B*40 with a reduced risk (OR = 0.74 [0.57–0.97], p = 0.03). These associations remained nominally significant when analyses were further adjusted for the *C6orf10* locus (OR = 0.75 [0.58–0.98], p = 0.04 for B*40 and OR = 1.98 [1.05–3.73], p = 0.03 for B*57).

## Discussion

The association of *HLA-C* with psoriasis was first proposed as early as 30 years ago [35]. However, until recently doubts remained as to whether *HLA-C* or a nearby gene was the locus responsible for the observed association. One of the difficulties contributing to this is the fact that the HLA region has a complicated and extended linkage disequilibrium pattern, while harboring many immune response genes in high density. Recently, sequencing and haplotype analyses studies have concluded that *HLA-C* is the major risk determinant of psoriasis within the HLA region, and HLA-Cw*0602 is the main risk allele. However, one immediate question arose: is *HLA-C* the only susceptibility gene in this region? This question turns out to be challenging because any other putative psoriasis predisposing genes would have a weaker effect than HLA-Cw*0602, and the analyses would be complicated by potential linkage disequilibrium with *HLA-C*. To control for the effects of *HLA-C*, it would be optimal to use the *HLA-C* genotype *per se*; however, in a large multi-center study such as the present one, molecular typing of the HLA alleles would not be readily available. In this paper, we used the CEPH phased data which contains the phased alleles at *HLA-A, -B, -C –DR* and *–DQ* as well as thousands of SNPs in this region as a reference sample to identify SNPs that can accurately predict the *HLA-C* alleles. These genotypes can then be used to perform analyses to search for other susceptibility genes. As our validation study illustrated, we were able to accurately predict the *HLA-C* alleles in the GAIN samples, in spite of it being composed of a diverse (though all white) mixture of individuals of differing European backgrounds. In addition to the present study, others have found similar utility of this approach across European-derived populations. For example, a validation assessment of the imputation method carried out on Dutch, UK, Spanish, and Italian samples showed high sensitivity and specificity in imputing *DQA1*, *DQB1* and *DRB1* alleles [33],[36]. Moreover, the genomic control [37] parameter of our samples was 1.03, suggesting that population stratification has negligible impact on our association results [32].

Through this imputation, we confirmed that HLA-Cw*0602 is the major psoriasis risk determinant within the HLA region, which has a much stronger association with psoriasis than the most significantly associated SNP, rs12191877. This has reinforced the importance of using *HLA-C* risk allele *per se* in the analyses of MHC, since adjusting for the surrogate SNP cannot fully control for *HLA-C*. Our analyses also provided further evidence that HLA-Cw*1203 is associated with psoriasis, although it is not clear whether HLA-Cw*1203 is a risk allele itself, or is in LD with the risk allele of another susceptibility gene near *HLA-C*. On the other hand, HLA-Cw*1203 was not associated with psoriasis in the Han Chinese after adjustment for HLA-Cw*0602. These may imply that HLA-Cw*1203 does not confer risk to psoriasis; the discrepancies in its association with psoriasis in different studies may be due to the different LD patterns among populations.

To search for additional loci for psoriasis in the MHC region, we conducted logistic regression analyses adjusting for the imputed HLA-Cw*0602, and identified two loci within 1.2 Mb of *HLA-C*, one around the *C6orf10* gene, and one between *HLA-B* and *MICA*. Both of these loci were significantly associated with disease risk after Bonferroni correction for the number of SNPs considered in the analyses. Furthermore, data analyses demonstrate that they are not simply reflecting linkage disequilibrium with *HLA-C*, since 1) the associations are not secondary to HLA-Cw*0602 as shown in the analyses adjusted for HLA-Cw*0602 or within the HLA-Cw*0602-negative subset of samples, 2) further adjustment for HLA-Cw*1203 in analyses did not substantially change our results, and 3) no other *HLA-C* major allele showed association with psoriasis in our data. More importantly, both associated loci were replicated in an independent Han Chinese dataset after adjustment for HLA-Cw*0602, even though the LD patterns in Chinese are quite different from those in the GAIN dataset. All these observations imply the existence of other psoriasis risk-determining genes within the MHC. When the putative susceptibility loci and HLA-Cw*0602 were included in a logistic regression model, they all remained significantly associated with disease, showing that these loci are not attributable to each other; therefore, within the MHC there are at least three genes conferring risk of psoriasis.

The first locus we identified is located 1.1 Mb centromeric of *HLA-C*, indicated by the SNP rs2073048. It is noteworthy that in our previous paper, using a forward selection technique, we also found some evidence of another SNP (rs2022544) close to this locus that was associated with Ps, but this study did not control for the *HLA-C* risk allele, but rather for its surrogate SNP [32], rendering the results subject to residual association of *HLA-C*. The SNP rs2073048 is located within the 4^th^ intron of an open reading frame *C6orf10*, 27 kb telemetric to *BTNL2* and 144 kb centromeric to *NOTCH4*. There have been previous reports of association of psoriasis with *AGER*, which is 183 kb telomeric of rs2073048, and with *HLA-DRB1* that is 211 kb centromeric of rs2073048. In the present study, analyses of imputed serotypes of *HLA-DRB1* did not support the involvement of *HLA-DRB1* in disease pathogenesis (data not shown). Furthermore, as noted before, there is a recombination hot spot centromeric to *NOTCH4* [38], reducing the possibility that the association at this locus is attributable to those genes located telomeric to the recombination hot spot (*NOTCH4*, *AGER*, etc.), although they still cannot be completely excluded. Thus, *C6orf10* and *BTNL2* are better candidate genes for this locus. The most associated SNP found in the GAIN dataset (rs2073048) after correction for HLA-Cw*0602 is located within *C6orf10*, and the SNP found in the Chinese dataset (rs28732201) is close to the transcription start site of *C6orf10*; therefore, *C6orf10* is one of the most important candidate genes at this locus. It has been observed that the transcription of *C6orf10* in keratinocytes can be triggered by TNF-α (Gene Expression Omnibus dataset number: GDS1289) [39], an important proinflammatory cytokine in the pathogenesis of psoriasis, although the function of the *C6orf10* product is not known. Nevertheless, other genes cannot be excluded; more haplotype and sequencing analyses will be required to pinpoint the risk-conferring variants at this locus.

The second locus we observed after adjustment for *HLA-C* and *C6orf10* is between *HLA-B* and *MICA*, located 117 kb centromeric to *HLA-C*, suggesting the potential association of *HLA-B* or *MICA* with psoriasis risk. In the Chinese samples, this locus was also indicated by logistic regression analyses adjusted for *HLA-C* and *C6orf10*, by a SNP located only 1 kb telomeric to *HLA-B*. More importantly, although the linkage disequilibrium patterns and the *HLA-B* tagging SNPs were different between Chinese and white populations, the same *HLA-B* serotypes were associated with psoriasis: B*57 with an increased risk and B*40 with a reduced risk. These are suggestive of the involvement of *HLA-B* in psoriasis etiology. The role of *HLA-B* in psoriasis immuno-pathogenesis might be similar to that of *HLA-C*, which has been shown to bind peptide motifs that are shared between the streptococcal M proteins and the wound-healing-associated keratins k16 and k17, thereby clonally expanding the pool of skin-directed autoreactive CD8+ T cells [40]. Another candidate gene for this locus, *MICA*, is a distant homolog of the MHC class I protein. It can be induced by cellular or metabolic stress in the epithelia, acting as ligands for the activatory T-cell receptor, NKG2D. In psoriasis, it has been shown that MICA is down-regulated in lesional skin compared with non-lesional skin (p = 0.007, Gene Expression Omnibus dataset number: GDS2518) [41]. The under-expression of the MICA protein might allow the unwanted cells to escape the cytolysis by NK or CD8+ T-cells, resulting in keratinocyte proliferation and the enhanced inflammation inherent to lesions of psoriasis. Other genes within this region still cannot be excluded by our analyses; more detailed studies of *HLA-B* serotypes and MHC haplotypes are required to further elucidate the association of this locus with psoriasis.

The study of associations in the MHC region is notoriously difficult due to the presence of many genes involved in immune and inflammatory processes as well as the extensive and complex patterns of linkage disequilibrium. Our use of a genome-wide panel of SNPs that included nearly 2000 SNPs within the MHC, a validated prediction method to determine with high probability the presence of known *HLA-C* risk alleles for Ps, and a large sample of psoriasis cases and controls allowed us to begin to tease out different effects on psoriasis risk within the MHC region. Our discoveries are replicated in independent samples from another race, reinforcing the evidence of our findings. In combination with the loci reported in our previous work [32],[42], and those yet to be identified from large-scale replication studies of thousands of loci arising from our own and other genome-wide association studies, we anticipate that substantial progress will be made in the coming months in explaining the genetic basis of psoriasis. Perhaps more relevantly, we anticipate that some of the genes identified will prove to be attractive therapeutic targets, leading to improved treatment for this disease.

In conclusion, we provide evidence that two loci within the HLA region in addition to *HLA-C*, one near *C6orf10* and one near *HLA-B*, are significantly associated with psoriasis, suggesting that within MHC there are at least three genes moderating susceptibility to psoriasis. However we fully recognize that additional studies including re-sequencing and detailed haplotype analysis will be required to elucidate the causal variants.

## Materials and Methods

### Subject recruitment

The initial genome wide association scan involved 1409 psoriasis patients and 1436 controls recruited from the University of Utah, the University of Michigan, and the Washington University at St. Louis, USA. All cases and controls were of European descent. Informed consent was obtained from each participant. In total, 1359 cases and 1400 controls with 447,249 SNP genotypes passed the quality control process. The average age at onset of psoriasis was 24.3 years with the majority of patients (1127, 82.9%) developing psoriasis before age 40. Additional details on subject characteristics and recruitment can be found in Nair et al [32].

The samples in the replication analyses were 1139 cases and 1132 controls used in the initial GWAS of psoriasis conducted at the Anhui Medical University, Hefei city, Anhui province, China. These samples were recruited from Han Chinese populations by multiple hospitals in China. More details about the studied samples are described elsewhere [34]. The local institutional review board at each site approved the study protocol.

### *HLA-C*, *HLA-B*, and *HLA-DRB1* genotype imputation

We used the phased HLA and SNP genotypes contained in the HapMap CEU panel (30 trios) and an additional set of 90 CEPH samples [33] to search for SNP combinations in linkage disequilibrium with specific HLA alleles, using an approach similar to that taken by de Bakker et al [33], except that whenever possible, a combination of 3 to 4 SNPs was used. In each of our GAIN samples, we inferred the haplotypes of these chosen SNPs and the corresponding haplotype probabilities using the PHASE program version 2.1 [43],[44], for case and control populations separately as suggested by Mensah et al [45]. The imputed HLA genotype containing an allele of interest was represented as the estimated number of copies of each specific allele, and was calculated by summing the probability of having that allele given a specific haplotype, weighted by the corresponding haplotype probability:
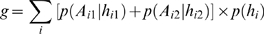
where *g* is the imputed number of HLA alleles, *A_i•_* denotes an event that the haplotype *i•* contains the HLA allele of interest, *h_i•_* is one of the two haplotypes of the haplotype assignment *i* from PHASE, and *p(h_i_)* is the haplotype probability. The *p(A_i•_|h_i_)* was directly obtained from the reference samples, and the *p(h_i_)* was calculated by the PHASE program. Thus, the uncertainties from the incomplete LD between the haplotype and the HLA allele, and from the haplotype reconstruction, were both integrated into the imputation; although not all uncertainty can be estimated due to the relatively small size of the HapMap and CEPH sample. To gain more power in association test, we minimized the overall uncertainty level (estimated by the averaged difference between the imputed and the most likely genotype), by maximizing the LD between the haplotype and the HLA allele, and by maximizing the haplotype probabilities by inclusion of nearby SNPs in low LD with the HLA imputing SNPs in haplotype reconstruction. These additional SNPs were selected by the HAPLOVIEW program [46], with a threshold of r^2^<0.1. For better imputation accuracies, a more stringent quality control strategy than the GWAS was applied, where those SNPs with <99.5% genotype call rates, or with evidence for departure from Hardy-Weinberg equilibrium at p<0.001 among controls, were not considered. To validate the imputed *HLA-C* genotypes, a sub-sample of 420 Utah psoriasis patients were genotyped using direct sequencing at Atria Inc. (South San Francisco, CA).

### Association analyses

e conducted trend tests to assess the association between SNPs and psoriasis, using the PLINK program [47]. To examine the association of a specific HLA allele with psoriasis, we used logistic regression analysis on the imputed HLA genotype, weighted according to genotype probabilities as suggested by Mensah et al [45], i.e., in our analyses of HLA, we used the imputed number of alleles (real number between 0 and 2), rather than the most likely number of alleles (0, 1 or 2) in logistic regression. To examine other SNPs within the HLA region, logistic regression was performed adjusted for *HLA-C* and associated SNP genotype, using the PLINK program [47]. Averaged odds ratio and the corresponding 95% confidence intervals for each additional number of minor allele of the studied SNP were calculated. Linkage disequilibrium plots and recombination rate plots were produced using the HapMap phase II data [48] by a C++ program written by the authors.
